# 

**Published:** 1785

**Authors:** 


### CATALOGUE.

1. AN Effay on the Puerperal Fever. By *Thomas Denman*, M. D. Licentiate in Midwifery of the College of Phyficians, and Teacher of Midwifery in London. The Third Edition. 8vo. *Johnfon*, London, 1785.

In the prefent edition of this valuable effay, though the whole may be faid to be enlarged, yet the point principally laboured is, the neceffity of a fpeedy application of fuch medicines as have been found ufeful in the cure and treatment of this fever, which the author confiders, in the beginning, as truly inflammatory.

2. A Treatife on the Glandular Difeafe of Barbadoes: proving it to be feated in the Lymphatic Syftem. By *James Hendy*, M. D. Member of the Edinburgh Royal Medical Society, Phyfician to His Majefty's Naval Hofpital at

Barbadoes,

Barbadoes, and Phyfician General to the Militia of the Ifland. 8vo. *Dilly*, London, 1784.

3. Remarks on the Difeafe lately defcribed by Dr. Hendy under the Appellation of the Glandular Difeafe of Barbadoes. By *John Rollo*, Surgeon in the Royal Artillery. 8vo. *Dilly*, London, 1785.

4. Obfervations on the Typhus, or low contagious Fever, and Means of preventing the Production and Communication of this Difeafe. By *David Campbell*, M. D. 8vo. *Johnfon*, London, 1785.

5. Obfervations on Antimonial Preparations; with a Defcription of a new Antimonial Powder, of peculiar Efficacy in Fevers and inflammatory Diftempers, and fingularly fuccefsful in the febrile Difeafes of Children. 8vo. *Herdffield*, London, 1785.

6. Tentamen Medicum de Aeroftatum ufu Medicinæ applicando. Auctore *Ludovico Leulier Duché*. 4to. Monfpelii, 1784.

7. Effai fur les Moyens de perfectionner les Etudes de Medecine, *i. e.* An Effay on the Means of improving the Study of Phyfic. By *S. A. D. Tiffot*, M. D. 8vo. Laufanne, 1785.

### INDEX.

A. Page

Acta Medicorum Suecicorum — — 334

Æther, Vitriolic, Obſervations relative to, — 53, 337

Aikin, Dr. J. his Edition of Dr. Lewis's Hiſtory of the Materia Medica, — — — — 89

———, Manual of Materia Medica, — 330

Air, Thoughts on the different Kinds of, — — 332

Almanacco di Sanita, — — 222

Antidotarium Coll Med. Bononienſis, — — 106

Antimonial Preparations, Obſervations on, — 443

B.

Balfour, Dr. F. on the Influence of the Moon in Fevers, 101

Baldinger, E. G. Sylloge Select. Opuſculorum, — 110

Banks, Sir Joſeph, Deſcription of a new Species of Cinchona, 177

Batch, A. J. G. C. Elenchus Fungorum, — 110

Bath, R. Eſſay on the Medical Character, — 102

Belladonna, Berries of, their Effects, — — 425

Bergman, Thorb. on the Uſefulneſs of Chemiſtry, — 99

Berkenhout, Dr. John, Symptomatology, — 101

Bertier, M. Traité d'Oſteologie, — — 112

Biſmuth, Magiſtery of, its good Effects deſcribed, — 406

Bloch, M. E. Von der Eingeweidewurmer, — — 76

Bonn, And. Deſcriptio Theſauri Hoviani, — — 223

Borſieri, J. B. Inſtit. Medicinæ practicæ, — — 112

Boys, William, Caſe of a Child who ſwallowed a Pin, — 400

Brodbelt, Dr. F. R. Caſes by, — 367, 371, 372

Brown, Joh. Elementa Medicinæ, — — 100

Brumwell, Mr. on the Effects of Belladonna, — 425

Butini, M. on the Gaſtric Juice, and Magiſtery of Biſmuth, 404

C.

Cæſarean Operation, by a Woman on herſelf, Caſe of, — 372

Caldanii Inſtit. Phyſiolog. curâ E. Sandifortii, — 103

Campbell, Dr. D. on the Typhus, — — 443

Camper, Petrus, de Calculo, — — 108

Camphor, its Effects in a Caſe of Mania, — — 120

———, remarkable Effects of, in a Dog, — 426

Caoutchouc, its generical Character, — — 315

Carex Arenaria, recommended as a Subſtitute for Sarſaparilla, 411

Caſſavi Root, its Uſe in Ulcers, — — 393

Cavendiſh, Henry, Experiments on Air, — — 171

Cawley, Thomas, Caſes by, — — 366

Chamberlaine, William, on Cowhage, — — 313

Chandler, Dr. B. on Apoplexies and Palſies, — 221, 324

Cheſton, R. B. on polypous Concretions of the Heart, 213, 225

Cinchona, a new Species of, deſcribed, — — 175

Cockell, Dr. W. on the retroverted Uterus, — 332

Coley, W. on an epidemic Ague, — — 221

Corp, Dr. W. on the Jaundice, — — 102

Cowhage, its Efficacy as an Anthelmintic, — 313

Cribb, William, on the Treatment of Hernia, — 259

Cullen, Dr. William, Firſt Lines of the Practice of Phyſic, 60

Cullum, T. G. Caſe of a Tumor between the Bladder and Rectum, 325

Davidſon,

D. Page

Davidson, George, on a new Species of Cinchona, — 175

Day, Thomas, on the Means of removing infectious Air, 101

Denman, Dr. Thomas, Essay on the puerperal Fever, — 442

Detmold, L. de Febre petechiali, — — 335

Dewell, Dr. T. Philosophy of Physic, — — 222

Dickinson, Dr. Caleb, on Fevers, — — 219

Dickson, J. de Plantis Cryptogamicis, — — 333

Digitalis purpurea, Accounts of its Effects, — 55, 145, 298

Dobson, Dr. M. on fixed Air, — — 332

—— the good Effects of a resuscitated Salivation, 419

Douglas, Dr. Andrew, Case of a ruptured Uterus, — 91

—— singular Cough, — 416

Duché, L. L. de Aerostatum usu medicinali, — 443

E.

Emmet, T. A. Oratio coram Soc. Phys. Edin. — 101

Eustachii, B. Tabularum Anatom. Explicationes novæ, - 223

F.

Fabricii, P. C. Animadversiones Medic. — — 111

Fæces, hardened, Case of, — — — 355

Faxe, A. on the Health of Seamen, — — 333

Fearon, Henry, on Cancers, — — — 100

Fernandez, F. B. de las Enfermedades de los Exercitos, 224

Fœtus, Extra-uterine, Cases of, — — 52

——, spontaneous Evolution of, Instances of, — 371

Ford, Edward, Case of a strangulated Hernia, — 118

Fordyce, Gulielm. Fragmenta Medica et Chirurgica, — 221

Fothergill, Dr. John, his Works published by Dr. Lettsom, 218

——, on the Epilepsy, — — 321

—— Sick Head-Ach, — 328

—— Cure of Fluxes, — 422

Fowler, Dr. Thomas, on the Effects of Tobacco, — 185

Fox Glove, see Digitalis.

Fragility of the Bones, Case of, — — — 288

G.

Gardane, M. des Maladies des Creoles, — — 107

Gardiner, Dr. John, on the Animal Oeconomy, — 101

Gastellier, M. des Specifiques en Medecine, — 112

Gastric Juice, its Effects as a topical Application, — 405

Gehler, C. de despiciendis Medicinæ irrisoribus, — 335

Geller, H. E. de Zinco, — — — 336

Gillespie, Leonard, on the Putrid Ulcer, — — 373

Goettling, J. F. A. Pharm. Chemischer Operationen, — 334

Goodwin, W. on a Fragility of the Bones, — — 288

—— Case of an encysted Tumor, — — 292

Gout, in the Stomach, removed by Æther, — 53, 341, 348

Grant, Alexander, on the Use of Opium, — 5, 130

Gruner, C. G. de Infanticidio, &c. — — 223

—— von kleinen med. Schriften, — 106

H.

Haighton, John, Case of Hydrophobia, — — 361

Hall, John, of an Aneurism of the Aorta, — 213

Hamilton, Dr. A. Outlines of Midwifery, — 219

——, Dr. R. on a Case of Fragility of the Bones, - 291

Harrison, J. Effects of fixed Air in Gangrene, — 331

Hatzfeld,

Page

Hatzfeld, F. G. de, de Anthropophago, — - 112

Haygarth, Dr. J. on the Prevention of Small-pox, — 206

Heart, Mal-conformation of, Cases of, 407, 433, 434, 436

Heineker, J. de Morbis Nervorum, — — 111

Hendy, Dr. James, on the Glandular Disease of Barbadoes, 442

Hernia, strangulated, Remarks on the Treatment of, 118, 259

Hoffman, his Anodyne Mineral Liquor, Preparation of, 351

Houlston, Dr. Tho. Case of a Man who swallowed Sublimate, 271

Humane Society, Reports of, —— — 331

Hunczousky, J. on the English and French Hospitals, - 222

Hunter, Dr. W. his Introductory Lectures, — 190

———————— Cases and Remarks by, — 430, 433, 437

Hussey, Dr. G. on Fevers, —— —— 330

Hydatids voided by the Mouth, Cases of, — 139, 293

Hydrocephalus, Cases of, — — 113, 214

——————, as it occurs in maniacal Cases, described, 159

Hydrophobia, Case of, — — — 361

J.

Johnson, James, Cases by, — 293, 295, 354, 355

Irving, R. Experiments on the Peruvian Bark, — 219

K.

Kidney, Enlargement of, Instances of, — 427, 428

King, S. C. Case of a Pen extracted from the Oesophagus, 426

Kirwan, R. Remarks on Mr. Cavendish's Experiments on Air, 174

Krumbolz, C. H. de Semine Mulieris, — — 336

Krzowitz, W. Trnka de, de febre hectica, —— 81

Kuhn, C. G. der Med. und Phys. Elektricitæt. — 334

L.

Lamure, M. de, Elemens de Matiere Medicale, — 168

Lausanne, Memoires de la Société de, —— 106

Leonardo da Vinci, his Anatomical Skill, —— 191

Lettsom, Dr. J. C. his Edition of Dr. Fothergill's Works, 2[illegible]8

Lewis, Dr. W. Exper. History of the Materia Medica, — 89

Limes, good Effects of, in Ulcers, — — 381

Lind, Dr. James, on the Effects of Æther in the Gout, 53

Linné, Car. v. on the Study of Nature, —— 333

Lloyd, Mr. Case of a Tumor on the Head, — 424

Lucas, James, on Cataracts, — —— 429

M.

Macquer, J. P. Dizionario di Chimica, —— 107

Magnetism, Animal, Works relative to, 102, 103, 104, 105, 112, 224

Mania, effects of Camphor in, 110

Malt, Infusion of, its good Effects in Pyuria, — 295

Marat, M. sur l'Electricité Medicale, —— 111

Marchan, M. sur la Cataracte, — — 109

Martyn, Thomas, Letters on Botany, —— 332

Mason, John, Cases of Dropsy cured by Opium, — 213

Maximinus, And. Novæ Explic. Tabul. Anat. B. Eustachii, 223

Medical Observations and Inquiries, —— 211, 321, 414

Meier, C. G. de Carice Arenaria, —— — 413

Merz, C. F. de Caricibus quibusdam, —— — *ibid.*

Metieke, J. F. de Borace, —— —— 336

Meza, C. J. T. de, Tractatio de Arte Obstetricandi, — 335

Michell, J. P. de Sympathia inter caput et partes Generationis, 102

Michelitz, Ant. de Causis Respirationis, —— 108

Mills, Samuel Gillam, Case of a Lady who swallowed a Pin, 36

Mitchel,

Page

Mitchel, G. Case of Incontinence of Urine, — 417

Monch, C. uber einige Arzneymittel, — 106

Moon, its Influence on morbid Bodies maintained, 101, 395

Mofeley, Dr. B. on Coffee, — — 331

———, Dr. J. on the Effects of Mercury in Hydrocephalus, 113

Moyle, Richard, Cases and Remarks by, — 52, 252, 257

Munning, J. Case of a Curvature of the Spine, — 258

Murray, J. And. Apparatus Medicaminum, — 109

——— ——— Opuscula, — — 223

Mynors, Robt. History of the practice of trepanning the skull - - - 220

N.

Newburg, S. de Acrimonia Urinosa, — 335

Nyberg, C. J. de Aeris fixi usu medico. — ibid.

O.

O Donnel, John, Case of a Man covered with Wens, — 33

Oliver, Dr. W. Effects of Camphor in Mania, — 120

Opium, its Effects in obviating morbid Irritability, — 5, 139

Otto, J. G. de usu Dulcamaræ, — — 336

P.

Paris, Memoirs of the Royal Academy of Sciences at, 179, 305

Pascoe, James, Case of a Tumor of the Leg, — 141

Payne, Thomas, Case of a Wound of the Throat, — 28

Pearson, Dr. George, Case of a diseased Kidney, — 426

———, John, Effects of Opium in a Suppression of Urine, 429

Pin, swallowed, Instances of, — — 36, 400

Piquer, Andre, Obras posthumas, — — 336

Place, F. de Diabete. — — — — 335

Polypus of the Heart, Remarks on, — — 225

———, bronchial, Case of, — — 252

Potter, T. his Medical Practice, — — 331

Poulle, Alexander, de Aere vitali, — — 108

Powell, J. Case of Hydatids voided by the Mouth, — 139

Proepffer, J. H. de Phthisi pulmonali, — — 335

Pugh, Dr Benjamin, on the Climate of Naples, &c. — 329

——— ——— Waters of Balaruc, — 331

R.

Ramazzini, on the Diseases of Tradesmen, German Transl. of, 110

Reinick, G. de Moscho, — — 336

Relaxation of the Body, Thoughts on, — 100

Relhan, R. Flora Cantabrigiensis, — 333

Renwick, W. his Addrefs to Parliament, — 221

Ribaucourt, P. de, sur l'eau minerale de Fruges, — 111

Richard, M. Caractere generique du Caoutchouc, — 315

Riollay, Dr. F. on the Doctrines of Hippocrates, — 86

Roederer, J. G. de Morbo mucoso, — — 110

Rollo, John, on the Glandular Disease of Barbadoes, — 443

Rubio, Ant. Pafqual y, del Garotillo, — 223

Rufpini, B. on an extraordinary Styptic, — 222

S.

Scarpa, Ant. de promov. Anatom. admin. rationibus, — 103

Schlegel, J. C. Collectio Opusculorum, — 334

Sequiera, Dr. J. H. Case of difficult Deglutition, — 414

Simmons, Dr. S. F. on the Hydrocephalus which occurs in Mania, 159

——— ——— Life of Dr. Hunter, — 218

Skeete, Dr. Thomas, Case of Effusion of Bile, — 274

Small,

| | Page |
|---|---|
| Small, Alexander, Observations on the Gout, — | 425 |
| Small pox, Essay on the Prevention of, ——— | 206 |
| Spallanzani, Abbé, his Dissertations, ——— | 330 |
| Spine, Case of a Curvature of, —— —— | 358 |
| Stack, Dr. R. Medical Cases, —— —— | 101 |
| Styx, M. E. de Nervo Crurali, —— —— | 336 |
| Sublimate, corrosive, large Quantity of it swallowed, — | 271 |
| Swediar, Dr. F. on the Venereal Disease, —— | 82 |
| **T.** | |
| Tacconus, Caj. Case of Mal-conformation of the Heart, | 407 |
| Tænia, systematic Division of the Genus of, —— | 78 |
| Taube, J. der Kriebelkrankheit, — — | 333 |
| Thouvenel, M. sur la Formation de l'Acide nitreux, — | 109 |
| Tickell, William, History of vitriolic Æther, —— | 337 |
| Tissot, M. sur les Etudes de Medecine, ——— | 443 |
| Tobacco, its medical Effects, —— —— | 185 |
| Transactions, Philosophical, —— — | 171 |
| Trye, C. B. on morbid Retention of Urine, —— | 222 |
| **V.** | |
| Vein, ruptured, Case of, —— —— | 141 |
| Vogel, S. G. Handbuch der praktischen arzneywissenschaft, | 109 |
| **U.** | |
| Underwood, Dr. M. on the Diseases of Children, — | 318 |
| Uterus, ruptured, Case of, —— —— | 92 |
| ———, inverted, Case of, —— —— | 367 |
| **W.** | |
| Warren, Dr. J. on the Use of Digitalis in Dropsies, — | 145 |
| Wathen, Jonathan, on the Cataract, ——— | 221 |
| Watt, James, on the constituent Parts of Water, — | 175 |
| Wauters, P. E. de Plantis Belgicis, ——— | 224 |
| Webel, C. G. F. de Sputis, —— — | 112 |
| Weisz, J. Pyretologia practica, —— —— | 223 |
| Wens, Case of a Man covered with, — — | 33 |
| Werner, P. C. F. de Vermibus Intestinalibus, —— | 110 |
| White, Thomas, on Scrophula, —— —— | 104 |
| Withering, Dr. W. on the Fox Glove, ——— | 298 |
| Worms, round, systematic Arrangement of, —— | 79 |
| Wright, Jer. Case of Extravasation of Blood into the Pericardium, | 211 |
| ———, Dr. W. on the Use of Cold Bathing in Tetanus, | 415 |

